# Impact of Obesity and Age on Mouse Corneal Innervation at the Epithelial-Stromal Interface

**DOI:** 10.1167/iovs.65.5.11

**Published:** 2024-05-06

**Authors:** Justin A. Courson, Rolando E. Rumbaut, Alan R. Burns

**Affiliations:** 1 Department of Medicine, Baylor College of Medicine, Houston, Texas, United States; 2 Center for Translational Research on Inflammatory Diseases, Michael E. DeBakey Veterans Affairs Medical Center, Houston, Texas, United States; 3 College of Optometry, University of Houston, Houston, Texas, United States; 4 Children's Nutrition Center, Baylor College of Medicine, Houston, Texas, United States

**Keywords:** obesity, aging, corneal nerves, electron microscopy, serial block-face

## Abstract

**Purpose:**

The corneal epithelium is the most highly innervated structure in the body. Previously, we reported a novel event whereby stromal axons fuse with basal epithelial cells, limiting nerve penetration into the epithelium. Although corneal–epithelial nerves undergo changes in sensitivity and distribution throughout life and in response to an obesogenic diet, it is unknown if neuronal–epithelial cell fusion is altered. Here, we sought to determine if neuronal–epithelial cell fusion frequency correlates with obesogenic diet consumption and age.

**Methods:**

Corneas were collected from C57BL/6 mice and evaluated for neuronal–epithelial cell fusion frequency using serial block-face scanning electron microscopy. To assess the correlation between diet-induced obesity and fusion frequency, 6-week-old mice were fed either a normal diet or an obesogenic diet for 10 weeks. To assess changes in fusion frequency between young and adult mice under normal dietary conditions, 9- and 24-week-old mice were used.

**Results:**

Mice fed a 10-week obesogenic diet showed 87% of central-cornea stromal nerves engaged in fusion compared with only 54% in age-matched controls (16 weeks old). In 9-week-old normal-diet animals, 48% of central-cornea stromal nerves contained fusing axons and increased to 81% at 24 weeks of age. Corneal sensitivity loss correlated with increased body weight and adiposity regardless of age and diet.

**Conclusions:**

Neuronal–epithelial cell fusion positively correlates with age and obesogenic diet consumption, and corneal nerve sensitivity loss correlates with increased body weight and adiposity, regardless of age and diet. As such, neuronal–epithelial cell fusion may play a role in corneal nerve density and sensitivity regulation.

The cornea is the transparent surface of the eye that covers the iris and pupil, and this transparency is necessary for our sense of sight. In addition, the corneal epithelium is the most highly innervated structure in the body, being 20× to 40× more densely innervated than tooth pulp and 300× to 600× more densely innervated than skin.^1^^–^^3^ The nerves of the cornea not only provide sensory information from the corneal surface but also play a major role in the general health and homeostasis of the cornea by providing trophic factors to resident epithelial cells.^4^^,^^5^ Innervation and organization of the cornea occurs early in development, with an extremely high density of innervation at birth that slowly diminishes throughout life, beginning as early as 15 years of age in humans.^6^ The process by which this reorganization is regulated is not well understood.^7^

Our published ultrastructural observations of mouse corneal nerves show a subset of central stromal nerves giving rise to axons that fuse with basal epithelial cells rather than passing into the corneal epithelium to contribute to the epithelial nerve plexus.^8^ Using serial block-face scanning electron microscopy (SBF-SEM) to image stromal nerves interacting with corneal epithelial cells at the epithelial basement membrane, we found that nearly half of all central corneal nerves showed signs of epithelial cell fusion. In contrast, peripheral corneal nerves penetrating the epithelial basement membrane did not show evidence of epithelial fusion.^8^ It was found that fused nerves were morphologically unique and easily distinguishable from penetrating axons. Fusing nerves had a smaller surface-to-volume ratio, indicative of large or swollen structures, and they were electron translucent and devoid of mitochondria at the fusion site when compared to penetrating nerves. Although the functional role of these fusion events is unknown, we speculate that nerve fusion with the epithelium may be a mechanism for limiting central corneal nerve density and sensitivity.

Loss of corneal innervation has been shown to diminish the barrier function of the corneal epithelium via ulceration or thinning of the surface epithelium while also slowing the healing rate of the epithelium post-wound.^9^ This can lead to stromal swelling or scarring, resulting in opacification of the cornea and loss of vision, or it can allow infectious agents into the deeper structures of the cornea and eye.^10^^,^^11^ Our previous work illustrates that the majority of age-related nerve density decline occurs from 3 to 6 months of age, representing the transition from young to adult mice.^12^ The age-related decline in corneal nerve density and sensitivity can also be accelerated by consumption of an obesogenic diet.^7^^,^^13^^,^^14^ Our recently published data show that corneal nerve density and sensitivity are diminished in mice fed an obesogenic diet when compared to age-matched mice fed a normal diet.^15^ By using this established mouse model of diet-induced obesity, we observed loss of corneal sensitivity after only 10 days and a marked reduction in corneal nerve density after 10 weeks of feeding mice an obesogenic diet. The mechanisms regulating obesity- and age-related reorganization in corneal innervation are unknown.

Understanding the ultrastructural changes that occur in corneal nerves as a result of obesity or age may aid in combating their respective corneal complications. For this reason, we sought to evaluate neuronal–epithelial cell fusion in mice exposed to an obesogenic diet, as well as during the transition from young to adult stages, where corneal sensitivity and nerve density are known to decline. Although the process by which corneal nerve reduction occurs is unclear, this study sought to determine if neuronal–epithelial cell fusion is correlated with the reorganization of corneal innervation that occurs as a result of the consumption of an obesogenic diet or as a function of age.

## Materials and Methods

### Animals

Male C57BL/6J mice were purchased from The Jackson Laboratory (Sacramento, CA, USA) and housed at the Baylor College of Medicine Children's Nutrition Research Center vivarium. All animals were handled according to the guidelines described in the ARVO Statement for the Use of Animals in Vision and Ophthalmic Research and the Baylor College of Medicine animal handling guidelines. All animal procedures were approved by the Animal Care and Use ethics committees at Baylor College of Medicine (IACUC number: AN2721) and the University of Houston (IACUC number: 16-005).

### Obesogenic Diet and Age

C57BL/6J mice were fed an obesogenic diet comprised of 41.31% kcal milk fat, 29.85% kcal sucrose, 11.8% kcal carbohydrate, and 15.26% kcal protein (diet #112734; Dyets Inc., Bethlehem, PA, USA) consistent with our previously published work.^15^ Male mice were used, as female mice do not exhibit metabolic syndrome when fed an obesogenic diet as compared to their male counterparts.^16^^,^^17^ The estrous cycle of female mice has been shown to alter hormone levels affecting the inflammatory response, providing a protective effect against weight gain and the development of diet-induced co-morbidities.^18^^–^^20^ Mice were fed the obesogenic diet for 10 weeks, at which time nerve density was expected to be reduced by ∼30% compared to normal diet controls.^15^ Control mice and mice in the 9-week-old and 24-week-old groups (young and adult, respectively) were fed a normal chow diet comprised of 14.79% kcal fat, 0.31% kcal sucrose, 61.81% kcal carbohydrate, and 23.09% kcal protein (Advanced Protocol PicoLab Select Rodent 50 IF/6F 5V5R; LabDiet, St Louis, MO, USA). Mice were allowed to age up to 9, 16, or 24 weeks before data and tissue collection.

### Evaluation of Mouse Weight and Adiposity

To evaluate body weight and adiposity, mice were weighed and then euthanized by CO_2_ asphyxiation followed by cervical dislocation to excise their epididymal adipose tissue (eAT). The epididymal fat pad is used frequently as a measure of visceral adiposity in obesity studies in male mice, as visceral adiposity is linked to the detrimental effects of obesity.^15^^,^^21^^–^^23^ The body adiposity index (BAI) was calculated as the ratio of whole-body weight to eAT weight (*n* = 8/group).

### Tissue Processing

Tissue fixation and plastic resin-embedding were performed as previously described.^24^^,^^25^ Briefly, following euthanization (as described above), enucleated eyes were placed in primary fixative (100-mM sodium cacodylate buffer, pH 7.2, containing 2.5% glutaraldehyde and 2-mM calcium chloride) for 2 hours at room temperature. Fixed corneas, with the limbus intact, were carefully excised from the enucleated eye and cut into four equal quadrants. These quadrants were then washed in buffer before sequential contrasting in osmium tetroxide–potassium ferrocyanide, thiocarbohydrazide, and then osmium tetroxide again, followed by uranyl acetate at 4°C overnight before being placed in a lead aspartate solution for 30 minutes at 60°C. Finally, the tissue was dehydrated through an acetone series prior to embedding in Embed-812 resin (Electron Microscopy Sciences, Hatfield, PA, USA) containing Ketjenblack EC600JD (Lion Specialty Chemicals Co., Tokyo, Japan) to reduce tissue charging.^26^ The block face was trimmed to a size of 1 mm × 1 mm, and the tissue block was then glued to an aluminum specimen pin and covered in silver paint to further reduce charging. Following the application of silver paint, a thin layer of gold was applied to the sample block using a Denton DESK II vacuum sputtering device (Denton Vacuum, Moorestown, NJ, USA) equipped with a standard gold foil target. The sputtering device was run for 2 minutes with a chamber pressure of 200 mTorr (argon gas) and 40 mA, resulting in an estimated 20-nm-thick gold coating.

### Serial Block-Face Scanning Electron Microscopy

Tissue blocks were sectioned using a 3View 2 system (Gatan, Pleasanton, CA, USA) mounted in a MIRA3 field emission scanning electron microscope (TESCAN, Pittsburgh, PA, USA), as previously described.^25^ Briefly, serial imaging was conducted under high vacuum (0.047 Pa) using a Schottky emitter and an accelerating voltage of 6 to 9 keV. Backscatter electron detection was used to image the block face. Beam intensity ranged from 5 to 7 on an arbitrary scale ranging from 1 to 20, spot size ranged from 4 to 7 nm, and a pixel dwell time of 12 µs/pixel was used for viewing during serial sectioning or 32 µs/pixel when capturing regions of interest for image analysis. The *z*-step distance between each serial image in these stacks was 100 to 200 nm. Magnification ranged from 3000× to 5500× and pixel size from 4 to 15 nm.

Imaging was conducted on central corneal samples. The central cornea was defined as having a diameter of 2 mm; the peripheral cornea, including the limbus, occupied the surrounding region (0.6 mm wide) immediately adjacent to the central cornea. The block face was viewed at low magnification to identify stromal nerves that approached the corneal epithelium prior to switching to high magnification to document nerve–epithelial interactions (i.e., penetration and fusion). Fusing nerve bundles were defined as large, electron translucent axons devoid of mitochondria that terminated at the basal epithelium. Multilamellar bodies were defined as organelles consisting of densely packed concentric layers of membrane. Nerves were assessed in 9-week-old mice fed a normal diet (*n* = 10 mice; 43 nerves), 16-week-old mice fed a normal diet (*n* = 5 mice; 63 nerves), 16-week-old animals fed a 10-week obesogenic diet (*n* = 8 mice; 92 nerves), and 24-week-old mice fed a normal diet (*n* = 6; 73 nerves). Each image series was an average of 50.7 ± 3.5 µm in length. A total of 0.011 mm^3^ was assessed in 9-week-old mice fed a normal diet, 0.015 mm^3^ was assessed in 16-week-old mice fed a normal diet, 0.014 mm^3^ was assessed in 16-week-old mice fed an obesogenic diet, and 0.018 mm^3^ was assessed in 24-week-old mice fed a normal diet.

### Post-Processing of Image Stacks

Image stacks were post-processed for spatial drift removal using Gatan DigitalMicrograph 2.31.734.0 software. Subsequent three-dimensional segmentation and reconstruction were conducted using Amira 6.0.1 software (FEI Company, Hillsboro, OR, USA). The contours of structures of interest were manually traced for each image in the image stack using a digitizing pen connected to a tablet (Wacom, Kazo, Japan). Traced profiles were used to produce three-dimensional volumetric reconstructions. The basement membrane was identified by its characteristic electron density on the stromal, posterior face of basal epithelial cells.

### Morphometric Analysis and Stereology

**Figure 1.**
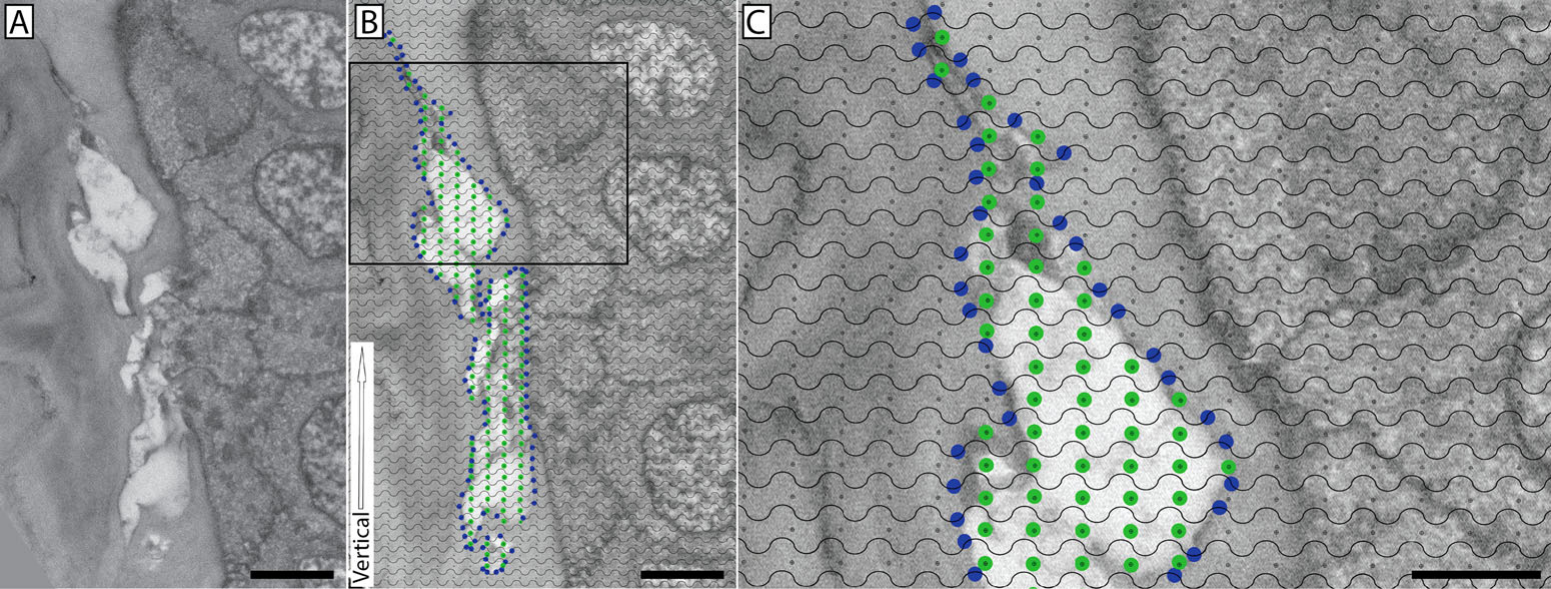
Stereological analysis of nerve surface-to-volume ratio in the central cornea using a cycloid grid. (**A**) A single random image from an SBF-SEM series shows a nerve bundle interacting with the basal epithelium. (**B**) A cycloid grid was randomly cast onto the image while maintaining the orientation of the grid (defined by the *vertical white arrow*) parallel to the epithelial basement membrane at the point of interaction. The cycloid grid contains lines and points, where line intersections are used to calculate surface and points are used to calculate volume. Points of nerve intersection with the cycloid grid lines are marked with *blue dots* (surface area), and grid points falling within the nerve bundle are denoted with *green dots* (volume). (**C**) Enlarged inset offers a magnified view of the grid. *Scale bars*: 5 µm (**A**, **B**) and 2.5 µm (**C**).

Morphometric analysis using standard stereological techniques was performed as previously described.^27^^,^^28^ To estimate the surface-to-volume ratio of fusing and penetrating nerves, a cycloid grid was used (Fig. 1). Briefly, serial electron images were obtained of both fusing and penetrating nerve events as they approached and interacted with the corneal basal epithelium. The serial images in which the nerve was visibly interacting with the epithelium were identified, and an image was selected at random from this subset of images for analysis. This was done by assigning each image a sequential numerical identifier and utilizing a random number generator for selection. Digital micrographs were analyzed using Photoshop (Adobe Systems, San Jose, CA, USA) with a cycloid grid and manual object counting function.^29^ The vertical axis of the grid was oriented in parallel to the basement membrane within each image to account for the anisotropic properties of the cornea. Line intersections with the nerve bundle of interest were counted, as well as target points located within the nerve bundle. To avoid counting line intercepts and target points within nerves located on the epithelial side of the basement membrane, a restriction line was drawn from one end of the basement membrane pore to the other, and nerve counts were only made on the stromal side. The ratio between line intersections with the nerve and target points within the nerve was used to calculate the cell surface density, or surface-to-volume ratio, using an established stereology formula:

$$\begin{array}{c} {\hat{S}}_{V}=\frac{2\cdot \sum_{i=1}^{n}I_{i}}{l/p\cdot \sum_{i=1}^{n}P_{i}} \end{array}$$

where *I* is the number of intersections between the grid lines and nerve bundle, *P* is the number of grid points falling within the target nerve, and *l*/*p* is the length of test line per grid point (corrected for magnification).^29^

### Transmission Electron Microscopy

Some excised corneas were collected and fixed overnight at 4°C in 100-mM cacodylate buffer (pH 7.2) containing 2.5% glutaraldehyde. Corneas were washed the next day in cacodylate buffer, post-fixed in 1% tannic acid, transferred to 1% osmium tetroxide, dehydrated in an acetone series, and embedded in the Embed-812 resin. Ultra-thin, 100-nm-thick sections were cut, collected on single-slot copper grids (formvar-coated and carbon stabilized), and imaged on a Tecnai G2 Spirit BioTWIN transmission electron microscope (TEM; FEI Company, Hillsboro, OR, USA). In addition, some tissue blocks prepared for SBF-SEM, containing verified neuronal–epithelial cell fusion and nerve penetration into the basal epithelium, were removed from the Gatan 3View 2 system and used to obtain 100-nm, ultra-thin sections for high-resolution TEM imaging.

### Esthesiometry

Corneal sensitivity was measured in awake mice with a Luneau Cochet–Bonnet Esthesiometer (Western Ophthalmics Co., Seattle, WA, USA) as described previously.^30^ Briefly, the extended esthesiometer filament tip was applied to the central corneal surface, and the length was incrementally shortened to increase filament stiffness and applied pressure. This was repeated until threshold pressure sensitivity was reached and a blink response was observed. The process was repeated three times in both eyes for all animals to obtain an average response (*n* = 8/group).

### Statistical Analyses

The Brown–Forsythe test was used to confirm that data analyzed using parametric statistics met the required assumptions (normal distribution, sample independence with equal variance between groups). To compare parametric variables (e.g., corneal sensitivity, mouse weight, eAT weight, BAI, surface-to-volume ratios), a two-way ANOVA test was used followed by Tukey's post hoc test to compare between conditions. Alpha was set at 0.05 for significance. To compare the frequency of neuronal–epithelial cell fusion across all conditions, a χ^2^ test was conducted, followed by a Fisher's exact post hoc test to compare between conditions. Prism 9 (GraphPad, Boston, MA, USA) was used for statistical analysis, and data are shown as the mean ± SEM.

## Results

### Consumption of an Obesogenic Diet and Increase in Age Led to an Increase in Total Body Weight, eAT Weight, and Body Adiposity

**Figure 2.**
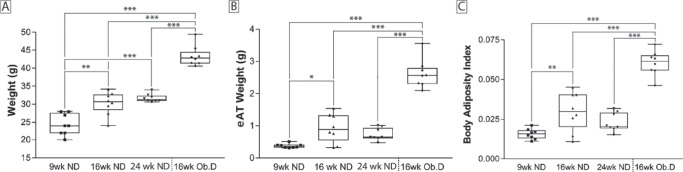
Effect of an obesogenic diet and age on weight and adiposity. The effect of age (9–24 weeks) was evaluated after feeding the mice a normal diet (ND), and the effect of an obesogenic diet (Ob.D) was evaluated in 16-week-old mice that had been fed the obesogenic diet for 10 weeks, beginning at 6 weeks of age. Mice were evaluated for changes in body weight (**A**), eAT weight (**B**), and body adiposity (**C**). *P > 0.05, **P < 0.01, ***P < 0.001.

As expected, mice fed a normal diet showed an age-related increase in total body weight, eAT weight, and body adiposity from 9 to 24 weeks of age (Fig. 2). Compared to 16-week-old age-matched control mice, these parameters increased by 30%, 150%, and 100%, respectively, when mice were fed an obesogenic diet for 10 weeks starting at 6 weeks of age (Fig. 2).

### Consumption of an Obesogenic Diet and Increase in Age Resulted in Loss of Corneal Sensitivity

**Figure 3.**
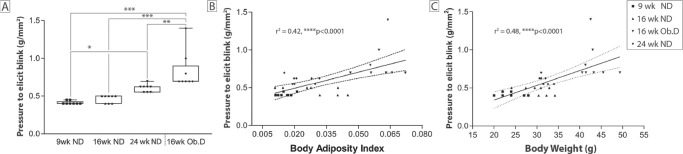
Changes in corneal sensitivity with obesity, age, and body adiposity. Corneal sensitivity (pressure needed to elicit a blink) was evaluated using a Cochet–Bonnet esthesiometer. (**A**) The effect of age (9–24 weeks) was evaluated after feeding the mice a normal diet (ND) or an obesogenic diet (Ob.D) which began at 6 weeks of age and continued for 10 weeks (16 weeks of age). (**B**, **C**) Pearson correlation plots of corneal sensitivity versus body adiposity and weight. *P < 0.05, **P < 0.01, ***P < 0.001, ****P < 0.0001.

Corneal sensitivity was evaluated using a Cochet–Bonnet esthesiometer (Fig. 3). An increase in the pressure required to elicit a blink indicates a loss of corneal sensitivity. There was a significant decrease in corneal sensitivity in 24-week-old animals compared to their 9-week-old counterparts, which was not apparent at 16 weeks of age. The 16-week-old animals that had been fed a 10-week obesogenic diet showed a marked decrease in corneal sensitivity when compared to their 16-week-old normal-diet controls. Furthermore, this loss of corneal sensitivity was significantly greater than the loss found in 24-week-old animals, despite being 8 weeks younger at the time of assessment (Fig. 3A). Regardless of the age and diet of the animal, loss of corneal sensitivity was significantly correlated (*P* < 0.0001) with weight and adiposity (Fig. 3B).

### Consumption of an Obesogenic Diet and Increased Age Led to an Increase in the Frequency of Neuronal–Epithelial Cell Fusion

**Figure 4.**
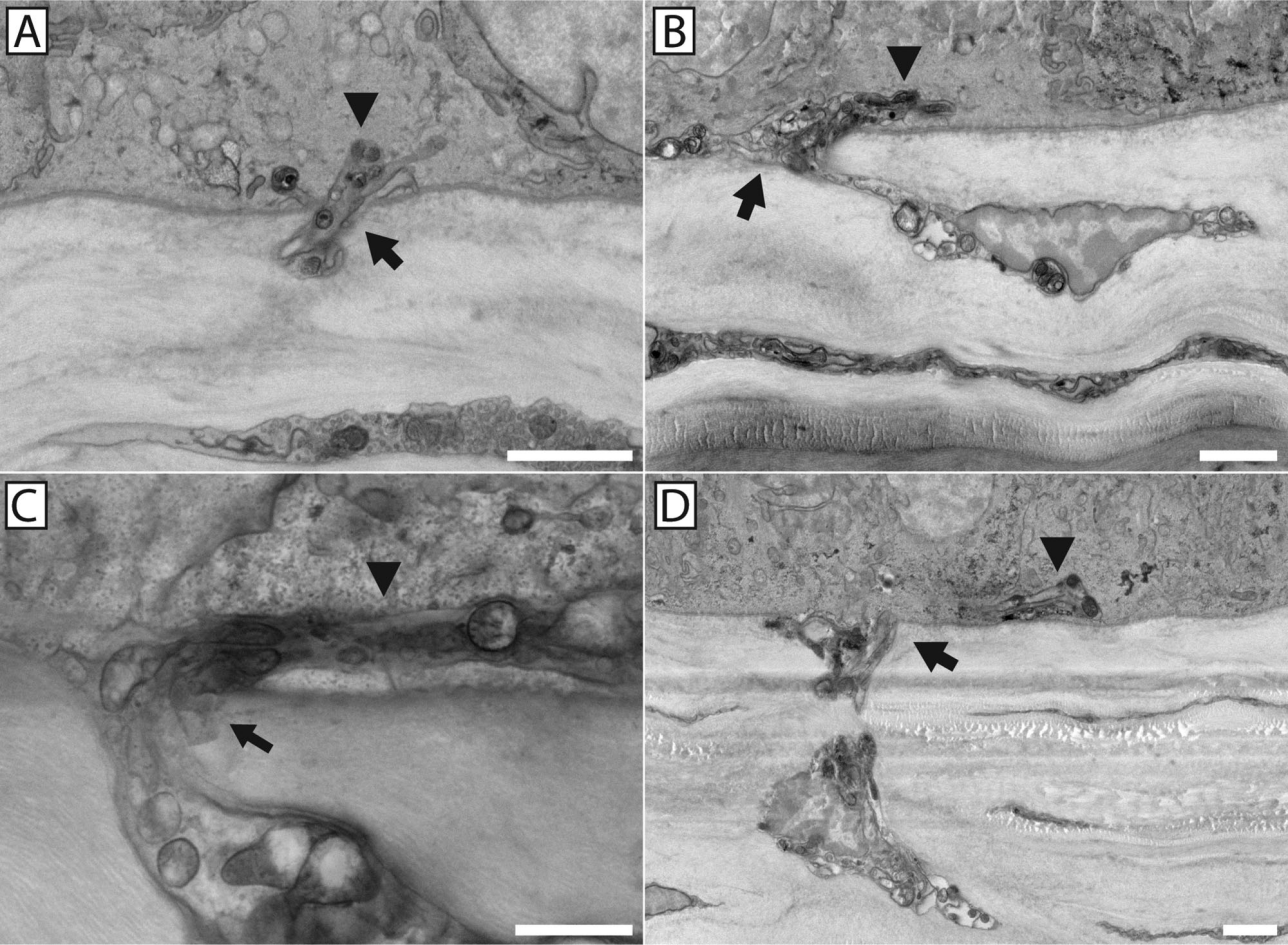
Nerve penetration through the basement membrane into the basal epithelial layer in the absence of neuronal–epithelial cell fusion. Examples of nerve penetration through the epithelial basement membrane in the absence of neuronal–epithelial cell fusion are shown. Nerves enter the epithelium through a discontinuity in the basement membrane (*arrows*), with a continuous basement membrane present on either side of the point of penetration. In all cases, portions of the penetrating axons can be seen successfully entering the basal epithelium (*arrowheads*). *Scale bars*: 2 µm (**A**, **B**, **D**) and 1 µm (**C**).

**Figure 5.**
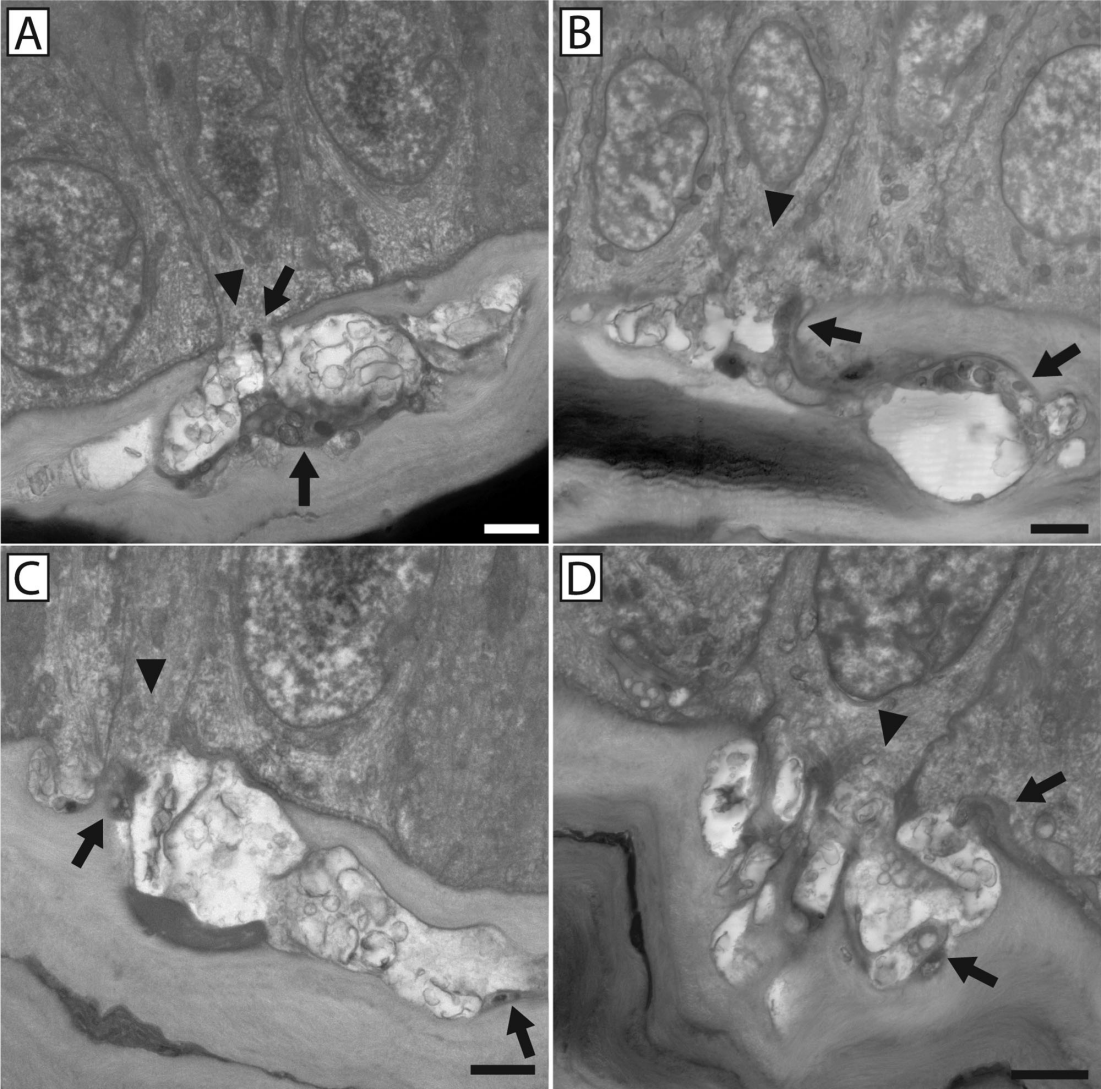
Neuronal–epithelial cell interactions involving both penetrating and fusing axons within the nerve bundle. Examples of neuronal–epithelial cell fusion at the stromal–epithelial interface are shown. In each event, a large electron-translucent portion of each nerve bundle can be seen fusing with the basal epithelium through discontinuities in the basement membrane (*arrowheads*). The electron-dense portion of the nerve bundle (*arrows*) successfully penetrates into the basal epithelium and in some cases can be seen enveloped by the basal epithelium and extending branches contributing to the subbasal plexus. *Scale bars*: 2 µm.

**Figure 6.**
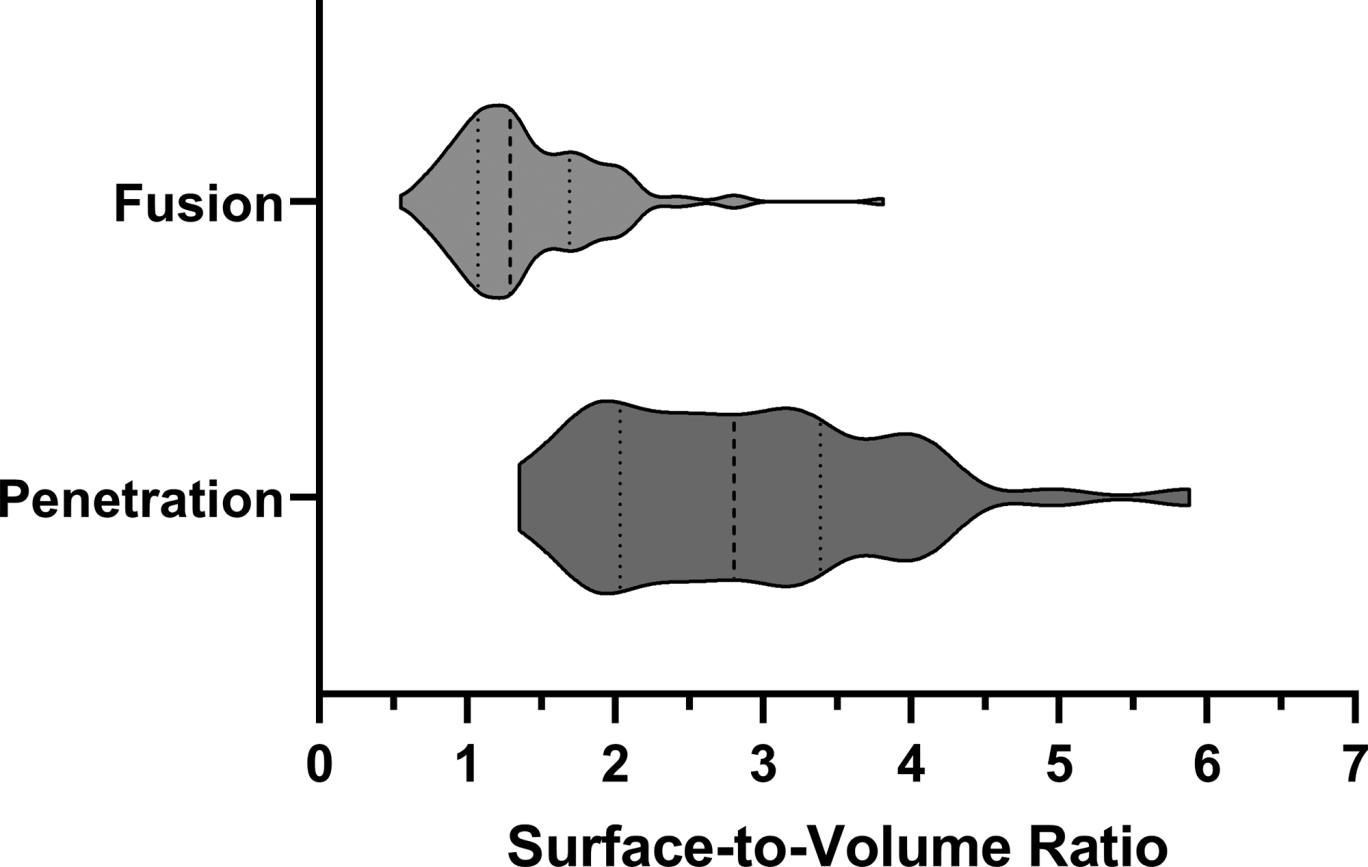
Stereological analysis revealed that nerves involving neuronal–epithelial cell fusion exhibit a decreased surface-to-volume ratio. The graph depicts the surface-to-volume ratios of corneal nerves penetrating the epithelial basement membrane. Mice that were 9, 16, and 24 weeks old were fed a normal diet, and Ob.D mice were fed a 10-week obesogenic diet beginning at 6 weeks of age. The *dotted lines* indicate first and third quartiles, and the *dashed line* indicates median. **P < 0.01, ***P < 0.001.

**Figure 7.**
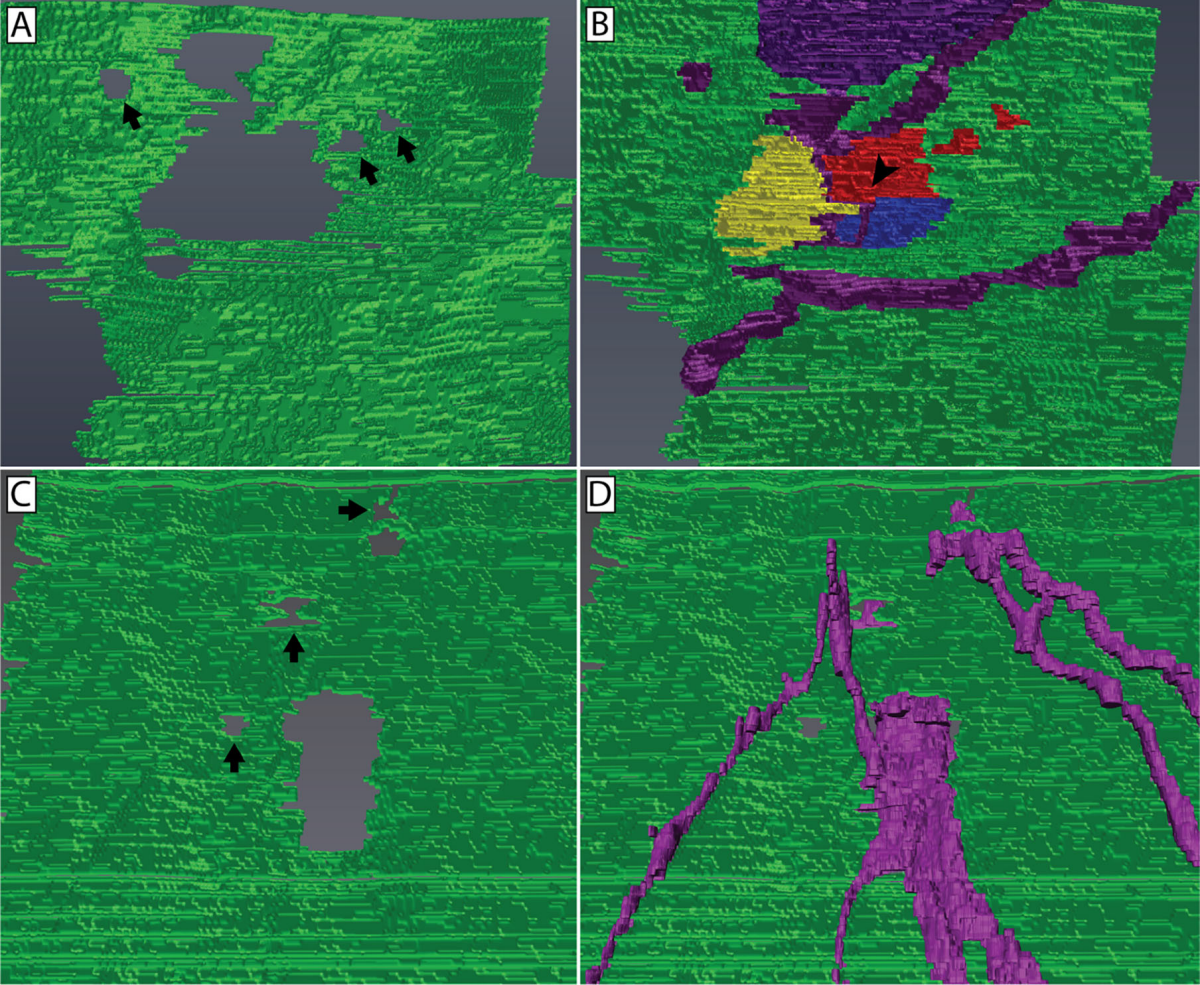
Three-dimensional reconstruction of basement membrane pores through which fusing and penetrating axons interact with the basal epithelium. Reconstructions of the basement membrane pores associated with a nerve bundle containing fusion (**A**, **B**) and a nerve bundle penetrating in the absence of neuronal–epithelial cell fusion (**C**, **D**) are shown. The majority of the basement membrane pore associated with a fusing nerve bundle is filled by the fusing axons (**B**). Here, we see the fusion between the nerve bundle and three separate epithelial cells (*red*, *yellow*, and *blue*) at a tricellular junction (*arrowhead*) at the level of the basement membrane (*green*). Penetrating axons (*purple*) can also be seen entering the epithelium. Small discontinuities in the basement membrane can be seen in close proximity to sites of nerve–epithelium interaction (*arrows*). Penetrating axons pass through a similarly sized hole in the basement membrane (**D**).

**Figure 8.**
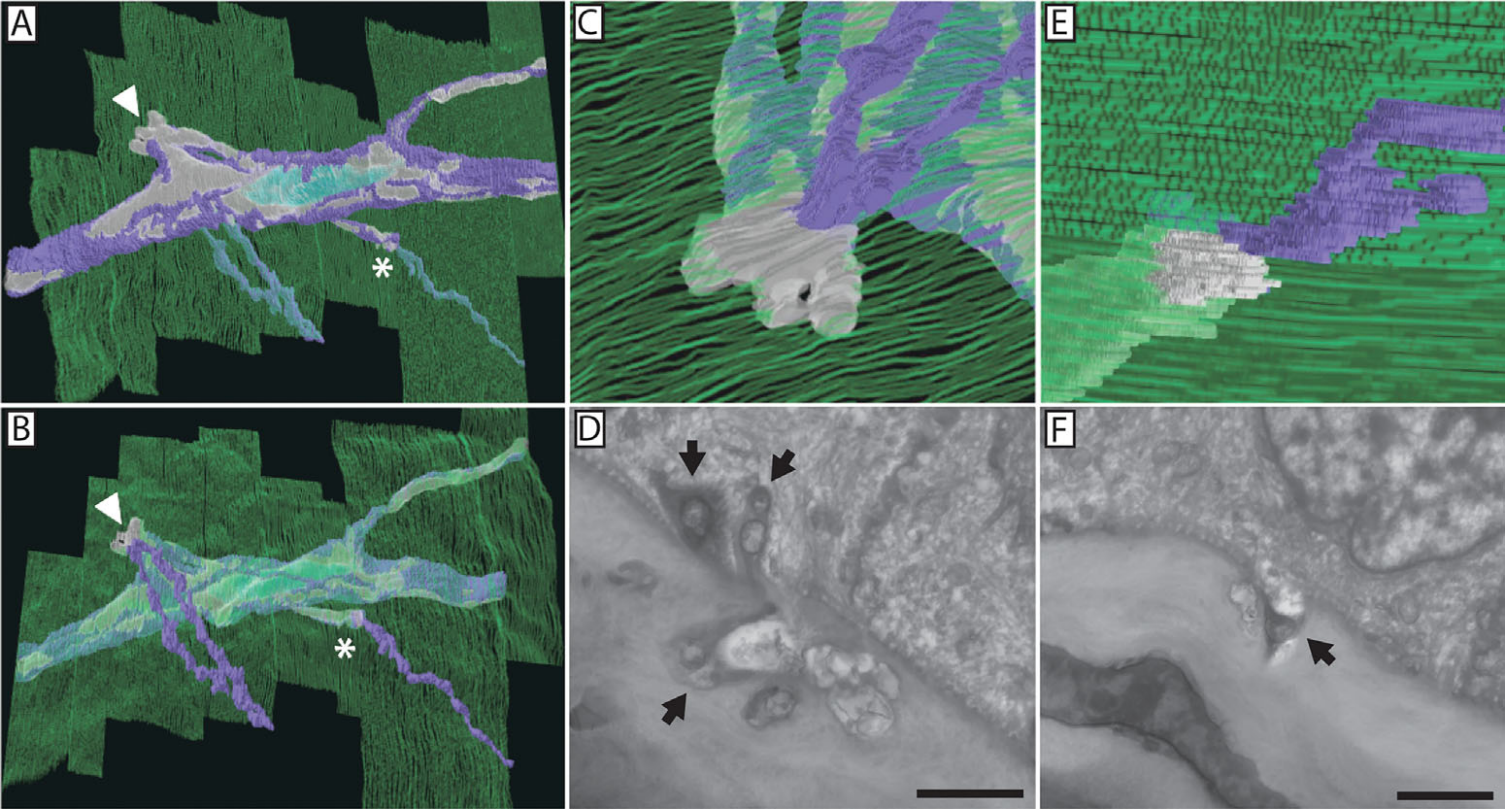
Three-dimensional reconstruction of a nerve bundle containing fusion reveals axons within the bundle that contribute to the subbasal plexus. (**A**) A single nerve bundle was imaged and reconstructed over a 50-µm distance containing two sites of nerve fusion, denoted by an *arrowhead* and *asterisk*. The nerve bundle is viewed from the stromal side of the basement membrane. The basement membrane is depicted in *green*. The electron-translucent, fusing portion of the bundle is depicted in *gray*, and the electron-dense, penetrating portion of the bundle is depicted in *purple*. A Schwann cell nucleus can be seen reconstructed in *turquoise*. (**B**, **C**, **E**) The nerve bundle is viewed from the epithelial side. (**D**) The first fusion site denoted with an *arrowhead* is represented at a higher magnification (**C**), in addition to a representative electron micrograph of the event. The second fusion site denoted with an *asterisk* is also represented at a higher magnification (**E**) in addition to a representative electron micrograph of the event (**F**). *Scale bars*: 2 µm.

Nerve bundles containing only penetrating axons (Fig. 4) were morphologically distinct from those containing fusing axons (Fig. 5). Fusing bundles contain axons that exhibit an electron-translucent “salt and pepper” axoplasm devoid of mitochondria and microtubules in the cytoplasm immediately surrounding sites of fusion, and they have a decreased surface-to-volume ratio indicative of a swollen cell (Figs. 1, 6). This morphological expression is compartmentalized exclusively to the axons that fuse; however, penetrating axons within the same bundle look characteristically dark and electron dense, with evident mitochondria and microtubules and a larger surface-to-volume ratio.^8^ Fusing axons terminate at the stromal–epithelial interface within pores in the basement membrane (Fig. 7), but penetrating axons pass into the basal epithelium and contribute to the epithelial nerve plexus (Fig. 8).

**Figure 9.**
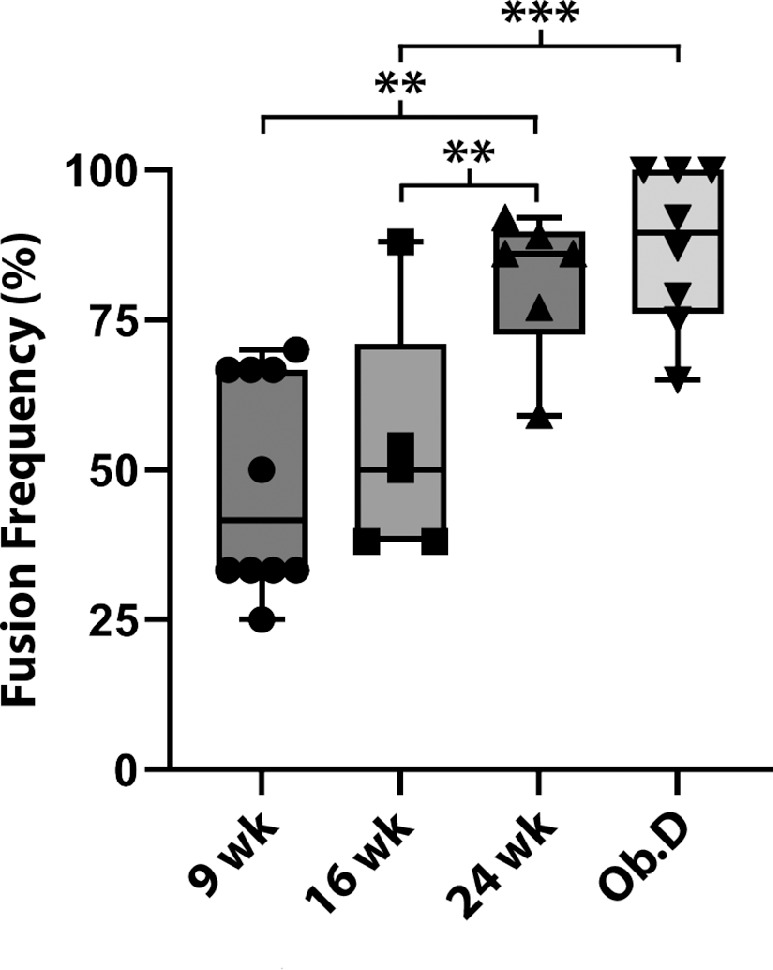
Frequency of neuronal–epithelial cell fusion increased with the introduction of an obesogenic diet and age. The effect of increased age on the frequency of neuronal–epithelial cell fusion was evaluated and plotted for mice fed a normal diet until they were 9, 16, or 24 weeks of age. The effect of introducing an obesogenic diet (Ob.D) on neuronal–epithelial cell fusion was evaluated in 16-week-old mice that had consumed the Ob.D for 10 weeks, beginning at 6 weeks of age. **P < 0.01, ***P < 0.001.

In mice fed a normal diet, the frequency of nerve fusion events was similar in 9- and 16-week-old mice, with 48% of nerves assessed containing axons that fused with basal epithelial cells compared to 54%, respectively. The 16-week-old mice that consumed an obesogenic diet for 10 weeks showed increased (*P* < 0.0001) nerve fusion events (87% frequency) when compared to 16-week-old mice fed a normal diet. Furthermore, this increased frequency of neuronal–epithelial cell fusion found in mice fed a 10-week obesogenic diet was not significantly different from the increase found in 24-week-old animals fed a normal diet (81% frequency), which was markedly elevated over both 9- and 16-week-old mice fed a normal diet (*P* < 0.001) (Fig. 9).

### Fusing Axons Are Electron Translucent and Swollen and Contain Accumulations of Autophagic Vesicles

**Figure 10.**
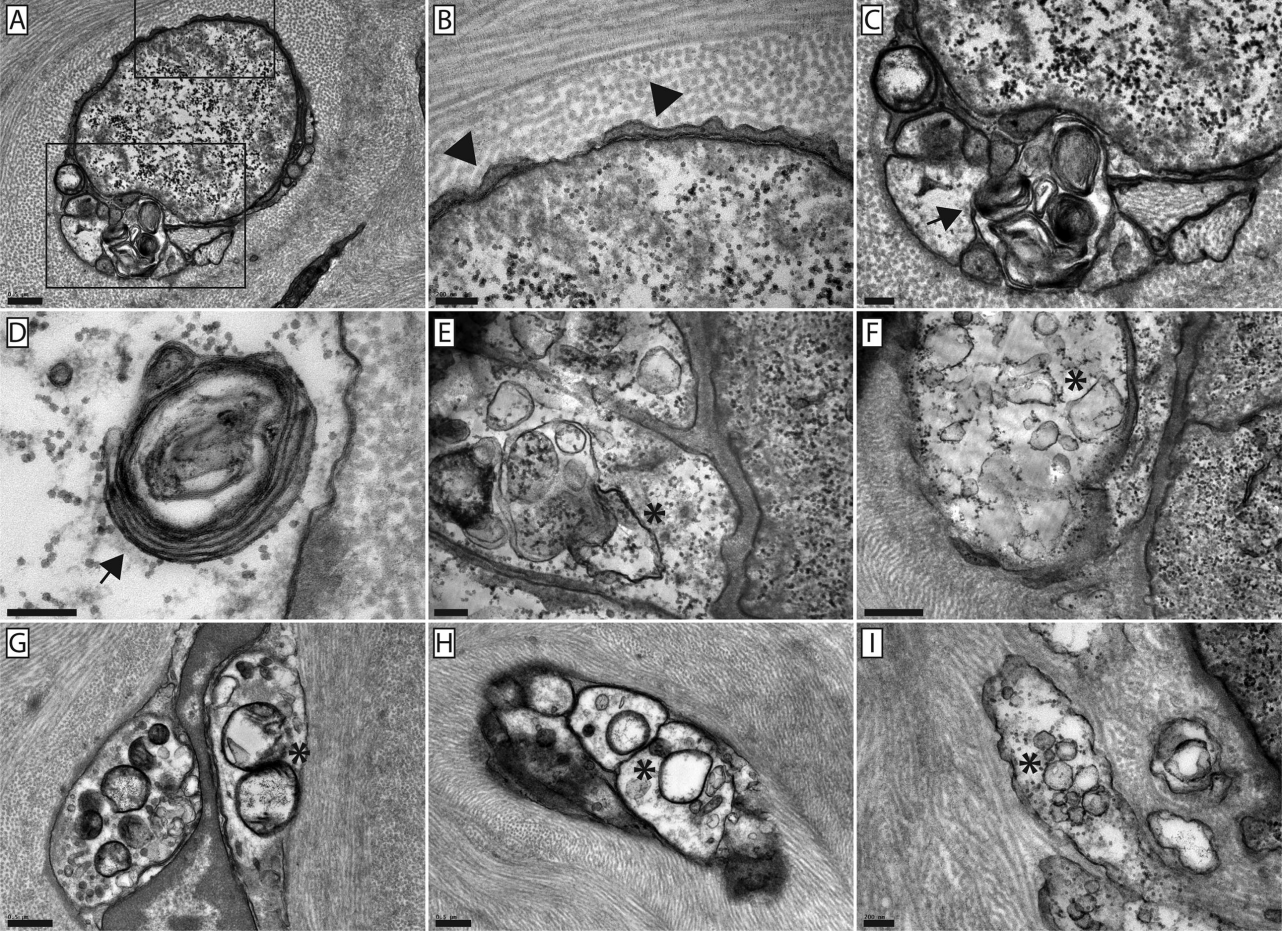
Autophagic vesicle accumulation proximal to points of neuronal–epithelial fusion. TEM images of nerve bundles that contain fusing axons, verified using SBF-SEM prior to TEM imaging. (**A)** A nerve bundle containing a swollen, electron-translucent axon as well as an additional electron-translucent axon profile containing an accumulation of autophagic vesicles. (**B**) Magnified view of the uppermost inset in **A**. The swollen portion of the nerve bundle is separate from the supporting Schwann cell. Here, thin Schwann cell projections can be seen wrapping the swollen, electron-translucent axon (*arrowheads*). (**C**) Magnified view of the lowermost inset in **A** where autophagic vesicle accumulation can be seen (*arrow*); note the absence of microtubules in the swollen, translucent regions of the nerve bundle. (**C**–**I**) Autophagic vesicles within fusing axons appear morphologically diverse, with multilamellar bodies (**C**, **D**, *arrows*), vesicles containing cytosol and degraded organelles (**E, F, I**, *asterisks*), and degenerating mitochondria (**G, H**, *asterisks*). All images were gathered from the obesogenic diet cohort. *Scale bars*: 500 nm (**A**, **F**–**H**) and 200 nm (**B**–**E**).

SBF-SEM and TEM imaging of fusing axons revealed the presence of autophagic vesicles at various stages of degradation within the swollen, electron-translucent axoplasm (Fig. 10). Autophagic vesicles accumulated close to sites of fusion and in some cases contained multilamellar bodies (Figs. 10A, 10C, 10D). Between 33% and 43% of fusing nerves were found to contain multilamellar bodies across conditions. Additional observations showed damaged and degenerate mitochondria in swollen and fusing axons (Figs. 10E–10I). As reported previously, TEM confirmed the lack of microtubules at points of autophagic vesicle and degenerate mitochondria accumulation, making it unlikely that retrograde transport and clearance of these structures occur.^8^

## Discussion

The purpose of this study was to determine if neuronal–epithelial cell fusion is associated with conditions characterized by loss of corneal sensitivity and nerve reorganization, such as the introduction of an obesogenic diet or with age. As expected, the data show that consumption of an obesogenic diet and age are associated with increased body adiposity and decreased corneal sensitivity. Furthermore, loss of corneal sensitivity correlated with weight and adiposity, regardless of age or diet. Interestingly, ultrastructural data collected using SBF-SEM indicate that neuronal–epithelial cell fusion is significantly increased in mice fed an obesogenic diet as well as during the transition from young to adult mice, concurrent with a significant loss of corneal sensitivity in both groups. Furthermore, these nerves contained autophagosome accumulations in close proximity to sites of neuronal–epithelial cell fusion. These data suggest that neuronal–epithelial cell fusion may contribute to nerve reorganization and loss of corneal sensation. These findings add a new level of complexity to the current understanding of corneal innervation.

Fusing axons appear to contain autophagosomes (Fig. 10).^31^^–^^33^ Autophagy is a lysosomal degradation pathway responsible for clearing damaged or aged cellular components from cells.^34^^–^^36^ As neurons are post-mitotic cells, they are particularly vulnerable to the buildup of unnecessary or dysfunctional organelles, damaged proteins, and cytotoxic material.^37^^,^^38^ Neurons rely heavily on autophagy to maintain cellular homeostasis and ensure their long-term viability and functionality, and baseline levels of autophagy are necessary for axonal homeostasis and survival.^38^ In studies where autophagosome activity was blocked, axons quickly degenerated, characterized by axonal swelling, not unlike what we observed with fusing neurons (Fig. 5), followed by retraction and, if autophagy is not restored, neuronal cell death.^39^^,^^40^ Interestingly, autophagic vesicles have also been linked with axonal pruning and axonal outgrowth.^41^^,^^42^ During axonal reorganization, autophagic vesicles are enriched in neuronal growth cones and within retracting regions, in particular.^42^^,^^43^ Furthermore, these studies show that autophagy is enriched in distal axons, such as the axonal projections present in the cornea. Given the dynamic nature of corneal innervation under homeostatic conditions, along with the increase in neuronal–epithelial cell fusion in response to an obesogenic diet and increased age, it is perhaps not surprising that the phenomenon of neuronal–epithelial cell fusion is associated with the presence of autophagic vesicles in corneal nerves. Indeed, it has been shown that generalized cell–cell fusion leads to enhanced autophagy.^44^^,^^45^ Whether autophagy plays a causative or mechanistic role in this unique cell–cell interaction is currently unknown.

The role of fusion between neuronal and epithelial cells is unknown. One possibility is that it might serve to enhance autophagy within corneal nerves. This may play a homeostatic role in young, healthy animals, as autophagy has been associated with robust neuronal regeneration under favorable conditions.^46^^,^^47^ However, in the presence of an obesogenic diet or during the transition from young to adult mice, a period in which the majority of age-related nerve density decline occurs, autophagy may be enhanced and dysregulated, leading to the loss of corneal innervation.^12^ Some of the early signs of axonal dystrophy include axon swelling and autophagy dysregulation and, because neuronal–epithelial cell fusion and its associated axonal swelling are upregulated in mice fed a 10-week obesogenic diet, it seems reasonable to posit that dysregulated autophagy plays a role in this pathology.^48^ Indeed, increased numbers of autophagosomes, indicative of dysregulated autophagy, are associated with pathology and neuronal death and have been implicated in the neuropathy that accompanies aging.^49^^,^^50^ Regardless, dysregulation of autophagy in corneal nerves deserves further study.

Previously published data from our lab show that corneal sensitivity is diminished and the normal corneal circadian rhythm of neutrophil influx into the limbus is blunted as early as 10 days into an obesogenic diet with a concomitant decline in nerve density, over-expression of corneal inflammatory mediators, and dysregulation of the wound-healing process following 10 weeks of obesogenic-diet feeding. Furthermore, this pathology precedes the onset of sustained hyperglycemia, a risk-factor for type 2 diabetes.^15^ The current study now finds that a 10-week obesogenic diet induces an upregulation in neuronal–epithelial cell fusion. Although the mechanism behind this induction is unknown, there are interesting cellular interactions of note in the cornea that might play a role in the diminished nerve density and sensitivity of the cornea as a result of an obesogenic diet. Our previous data show a blunting of the circadian accumulation of neutrophils in the corneal limbus following an obesogenic diet.^15^ It is well established that neutrophils are potent sources of vascular endothelial growth factor, a critical growth factor for the health of the vertebrate nervous system that promotes neurogenesis, neuronal patterning, neuroprotection, and glial cell growth.^51^ It is possible that the dysregulation of the homeostatic neutrophil response plays a role in the increase in neuronal–epithelial cell fusion and the loss of corneal nerve density and sensitivity that result from obesogenic diet consumption and during the transition from young to adult animals. Additionally, resident macrophages and dendritic cells have been shown to be activated by obesity.^52^^,^^53^ This is important to note, as changes in these populations correlate with diminished nerve density and delayed nerve regeneration.^54^^–^^57^

Although neuronal–epithelial fusion is more frequent in 24-week-old mice and autophagic vesicle accumulation increases with age, it is important to note that weight gain is a component of the natural aging process.^58^^,^^59^ This is of particular interest, as increased frequency of neuronal–epithelial cell fusion is positively correlated with increased body weight and adiposity. Furthermore, the accumulation of autophagic vesicles, as observed in fusing axons, correlates with age-related changes to metabolism, and the regulation of autophagy has been shown to change in response to environmental cues such as changes in nutrient levels within the body.^60^ Although these changes can occur in response to diet, they also correlate with normal age-related processes. As such, it is possible that the changes seen in the adult mice of this study result from the increase in weight and adiposity that occurs as mice mature. This is an intriguing proposition that deserves further study, as aging is correlated with the accumulation of reactive oxygen species which can lead to cell damage and senescence, as well as mitochondrial and DNA damage, all of which are associated with dysregulation of autophagic processes.^61^ Regardless, the extent to which obesity plays a role in increasing neuronal–epithelial cell fusion in mouse corneas as animals increase in age warrants further study.

The literature linking obesity to peripheral neuropathy provides an interesting context for the data presented here. Although obesity has been shown to lead to metabolic syndrome, low-grade systemic inflammation, and peripheral neuropathy, studies show that bariatric surgery may significantly reduce these pathologies.^62^^–^^64^ Obesity-related neuropathy is believed to result from mitochondrial dysfunction, oxidative stress, and inflammation with a significant decrease in corneal nerve density and sensitivity.^65^^–^^67^ However, bariatric surgery was shown to significantly improve neuropathy symptom scores and improve corneal nerve parameters such as nerve fiber density, corneal nerve branching, and nerve fiber length, indicative of nerve regeneration.^62^^,^^68^ Markers of low-grade inflammation, such as elevated serum IL-6 and high-sensitivity C-reactive protein (hs-CRP), are also diminished after bariatric surgery, alongside lower levels of triglycerides and higher levels of high-density lipoprotein.^62^ Given these striking benefits of bariatric surgery, it is also possible that dietary intervention may be enough to reverse corneal neuropathy and decrease the frequency of neuronal–epithelial cell fusion events in the cornea. Indeed, our previous works suggest dietary intervention may play a role in correcting corneal nerve dysfunction following the consumption of an obesogenic diet.^15^^,^^69^ It may be that there is a mechanistic link between obesity and diminished corneal health and that reducing obesity leads to improvements in corneal health.

## Conclusions

In conclusion, the data show that neuronal–epithelial cell fusion is positively correlated with loss of corneal sensitivity in mice fed an obesogenic diet, as well as during the transition from youth to adulthood. As such, we speculate it may play a role in nerve reorganization and loss within the cornea. Under normal conditions, a baseline level of neuronal–epithelial cell fusion may play a homeostatic role allowing for the pruning and regrowth of neuronal projections into the corneal epithelium, especially in the corneal center, where the corneal epithelium and nerve plexuses are known to be dynamic and continually shifting throughout life. In the presence of an obesogenic diet or with increased age, the rate of neuronal–epithelial cell fusion increases, alongside a loss in nerve density and corneal sensitivity. A greater understanding of the process of neuronal–epithelial cell fusion might pave the way for the creation of novel treatments for corneal neuropathy.
